# A simple model for predicting the signal for a head‐mounted transmission chamber system, allowing IMRT in‐vivo dosimetry without pretreatment linac time

**DOI:** 10.1120/jacmp.v15i4.4842

**Published:** 2014-07-08

**Authors:** Daniel Johnson, Steven J. Weston, Vivian P. Cosgrove, David I. Thwaites

**Affiliations:** ^1^ Department of Medical Physics and Engineering St James' Institute of Oncology, Leeds Teaching Hospitals NHS Trust Leeds UK; ^2^ Institute of Medical Physics School of Physics, University of Sydney Sydney Australia

**Keywords:** *in vivo*, transmission detector, radiotherapy, IMRT

## Abstract

The DAVID is a transparent, multi‐wire transmission‐style detector that attaches to a linear accelerator (linac) collimator for use as an *in vivo* detector. Currently, the normal method for using the DAVID is to measure a signal at the time of phantom‐based pretreatment verification and use that signal as a baseline to compare with *in vivo* measurements for subsequent treatment fractions. The device has previously been shown to be both stable and accurate.^1^, ^2^ This work presents the development of a predictive algorithm for the expected signal, eradicating the need to spend time on the linac prior to treatment, and thereby making the process more efficient. The DAVID response at each wire is a consequence of both primary radiation, from the leaf pair associated with the wire, and scatter radiation as a result of radiation incident on other parts of the detector scattering in the Perspex plate. The primary radiation was shown to be linearly proportional to both leaf separation and delivered monitor units (MU). The scatter signal dropped off exponentially with regard to distance. Both of these effects were modeled; the resulting algorithm was used to predict the response from ten five‐field IMRT head and neck plans. The system predicted all DAVID signals to within 5%, and was able to detect artificially generated changes in linac output. Having shown that the algorithm works, a new working paradigm is suggested, and the errors that can be detected are outlined.

PACS number: 87.55.N

## I. INTRODUCTION

Delivery of radiotherapy treatments using advanced techniques is widespread and involves the use of complex fluence and dose distributions. In response to several incidents that resulted in significant, unplanned exposure, a number of publications have suggested that online verification is a prudent step to ensure correct delivery and maintain public assurance. In some countries, *in vivo* dosimetry is recommended; in others, it is mandatory.^3^, ^4^, ^5^, ^6^

**Figure 1:**
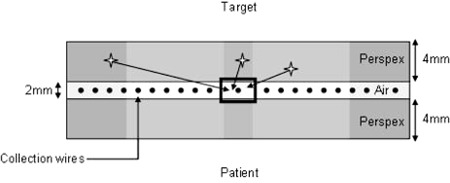
Cross section of DAVID device. The stars represent Compton events.

The Device for Advanced Verification of IMRT Deliveries (DAVID) (PTW, Freiburg, Germany) is a transmission‐type detector which can be attached to the linac collimator to measure output. The device consists of two polymethyl methacrylate (PMMA) plates separated by a 2 mm air gap. Inside the air gap, a series of collection wires run the length of the detector in the direction of MLC movement. There is one wire for each leaf pair, and each wire aligns with the center of the projection made by its corresponding leaf pair, making the device specific to the model of MLC with which it is intended to be used. The DAVID is discussed in detail by Poppe et al.;^(1)^ the only difference being that the current model does not have the guard wires discussed in that publication (Fig. 1). Transmission detectors, like the DAVID, attenuate the beam and will always require an increase in MU when in place (4% in the case of the DAVID^(1)^); furthermore, they do not assess the accuracy of the patient setup. The DAVID, unlike most *in vivo* techniques or devices, can detect ~ 1 mm inaccuracies in leaf position.^(2)^ Once attached, given that the DAVID is optically transparent, the device can stay in place during the whole fraction and series of treatments, making it a feasible option for the routine online verification of IMRT and VMAT treatments.

The current standard paradigm when using the DAVID involves the treatment being verified prior to the first fraction, using external dosimetry equipment and phantoms with the DAVID also in place. If the verification is successful, the DAVID signal collected during the verification is used as a baseline for subsequent treatment fractions. During treatment, the signal from each wire is collected *in vivo* for each beam or each segment (for 3D conformal and IMRT, respectively) and compared to the equivalent measurement made during the verification. If the readings are within a user‐defined range, then the treatment is deemed to be acceptable for that fraction. It has been shown that, through iterative deconvolution of the signals, the fluence passing through each MLC pair can be calculated.^(2)^ Comparison of treatment fluences with verification fluences has been shown to be more accurate than signal comparison, as the ambiguity in signal origin, arising from the scatter inside the PMMA, is reduced. (H.K. Looe, personal communication concerning the origin of the increased accuracy of the DAVID in his publication,^(2)^ February 13, 2012.)

Treatment verifications for IMRT and VMAT plans are conventionally performed by transposing the treatment plan on to a phantom, delivering the treatment to the phantom, and measuring the delivered dose. In addition to this, an MU check is performed by independent software on the treatment plan and clinical structure set. If the dose measured in the phantom agrees with that calculated by the planning system, within predetermined tolerances, and the independent dose check matches the TPS dose, the plan is deemed verified. This method verifies the TPS dose calculation, data transfer, and linac delivery. Though thorough, the process requires significant resources; transposing and recalculating plans takes time in the treatment planning department; and setting up the phantom and delivering the treatment requires time on the linac. There has been discussion in the literature as to whether this time‐consuming procedure is still necessary, for every patient, once confidence with a certain technique is built up.^(7)^ Other publications have questioned whether accepted methods of performing pretreatment verification reflect clinically relevant sources of error in the treatment plan.^(8)^

**Figure 2:**
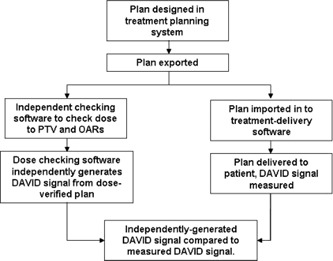
Suggested paradigm for *in vivo* dosimetry using the DAVID.

The aim of this work is to characterize the response of the DAVID in terms of primary and scatter radiation with regard to the leaf separation and delivered MU. This information is then used to develop a lateral response function that can be used to predict the DAVID signals, given the RT DICOM plan. This will allow the user to validate a plan with independent MU verification software, and generate a DAVID signal without spending pretreatment time on the linac or time recalculating dose on a phantom (Fig. 2).

## II. MATERIALS AND METHODS

### A. Equipment

All results were collected on a Synergy linear accelerator (Elekta AB, Stockholm, Sweden) equipped with an MLCi2 collimator.

All treatment plans have been delivered as clinical treatments in the department and were generated by the Monaco 3.0 TPS (Elekta AB, Stockholm, Sweden)

The DAVID is a transmission style detector, specific to the linac (MLC) model. As the MLCi2 collimator has an 80 leaf (40 leaf pairs) MLC, the DAVID used in this work had 40 wires. These collection wires are held in a 2 mm thick vented air gap that is encased by two polymethyl methacrylate (PMMA) plates, each 4 mm thick. On the inside of the PMMA, a thin layer of aluminum has been evaporated onto the inner surfaces. This layer is thin enough so that the device remains optically transparent, but thick enough to maintain a potential of 400 V between the plates and the collection wires. Ionization charge in the air gap, as a consequence of primary and scattered radiation, migrates towards the collection wires under the influence of the potential, each wire having a collection area of 0.03 cm^3^ per centimeter of length (Fig. 1).^(1)^

### B. Characterization of signal behavior

**Figure 3:**
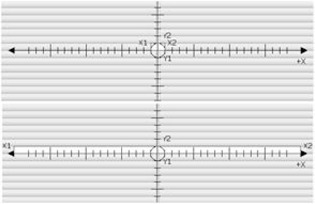
The central leaf pair was opened from a separation of 20 mm (top) to 400 mm (bottom) through incremental separations of 40, 70, 100, 150, and 300 mm. Through each separation, 5, 10, 25, 50 and 100 MU were delivered.

**Figure 4:**
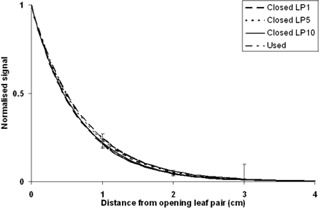
The normalized and averaged response of the central four wires taken from the same experiment that produced Fig. 4. The scatter radiation at distance >4 cm is too low to be detected.

The following was done in order to characterize the signal behavior:

- A single leaf pair was opened symmetrically about the central axis.
- Leaf separations of 20, 40, 70, 100, 150, 200, 300, 350, and 400 mm (projected at isocenter) were used.
- Through each separation, 5, 10, 25, 50 and 100 MU were delivered (Fig. 3).
- This test was performed using the central leaf pair, as well leaf pairs 5 and 10 (from center).
- The results for each delivery were normalized to the signal from the wire associated with the extending leaf pair.
- The normalized results for the range of extensions associated with a leaf pair were averaged and used to define the lateral response.
- The results were fitted with an exponential trend line; the associated exponents are shown in Table 1.
- These tests were repeated three times and a standard uncertainty of 1% was found, which was combined with the standard deviation of the normalized results to get the uncertanties displayed on Fig. 4.
- The lateral response exponent (Table 1) used in the predictive algorithm was found by iteratively comparing predicted results with measured results for a single treatment for multiple exponent values. The optimal exponent determined via this method was found to agree with the measured values (Fig. 4).

**Table 1:** The leaf pairs and their corresponding exponent

*Leaf Pair*	*Exponent*
1	−1.5515
5	−1.4468
10	−1.5106
Used	−1.4034

### C. Implementation and testing

The DAVID signal was independently collected three times for ten five‐field head and neck step‐and‐shoot IMRT treatments. All the treatments were previously used clinically, so passed the 3%/3 mm gamma criteria at a level >95% when subject to 3D dose verification. The results were averaged to account for variations in the DAVID response and linac delivery. These average responses were compared with the signal predicted by the developed method.

## III. RESULTS

### A. Characterization

**Figure 5:**
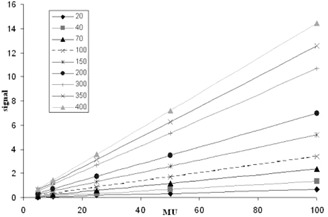
The response of the DAVID's central wire in arbitrary units for increasing MUs repeated for several different leaf pair separations (shown in the legend). All other leaves were closed throughout.

**Figure 6:**
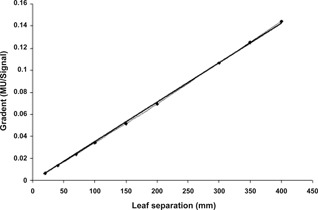
The gradients of the response taken from Fig. 5 plotted as a function of leaf pair separation.

The signal as a function of MU for various leaf pair separations is shown in Fig. 5. The change in the signal/MU gradients, as a function of separation distance, is shown in Fig. 6. The lateral response of the detector, as a result of scatter inside the DAVID's PMMA plate, for three leaf positions, is shown in Fig. 4.

The signal was observed to vary linearly with MU (Fig. 5) and the gradient of each plot was seen to vary linearly as a function of leaf pair separation (Fig. 6).

The results collected for the four wires either side of the central wire (eight wires altogether) were normalized to the value recorded by the central wire. The normalized results for each wire were averaged and plotted as a function of distance from the central wire. The lateral response of the DAVID was observed to be exponential in character (Fig. 4) and is a consequence of the scatter inside the PMMA plate on the beam side of the device.

**Figure 7:**
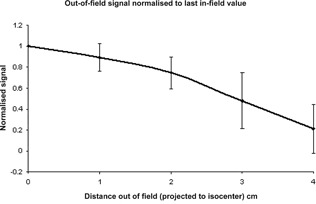
The normalized penumbral drop off as a function of the predicted signal from the first wire in the field. By using the predicted field value, the predicted penumbral signals use information that relies on the scatter in the field.

The field edge is defined by the position of the jaw in the X direction (the direction of leaf movement), wires in this region are subject to primary radiation that is penumbral in nature and scatter radiation; this is difficult to model in detail. For this simple model, the predicted signal for the first four out‐of‐field wires was defined as a function of the last in‐field wire. For example, in the case of wire 7 being in the field, wire 8, 9, 10, and 11 would be defined as $P_{1}\times \text{signal}$ at 7, $P_{2}\times \text{signal}$ at 7, $P_{3}\times \text{signal}$ at 7, $P_{4}\times \text{signal}$ at 7, respectively, where $P_{1-4}$ are fractional values defined by the function shown in Fig. 7. The values used for Fig. 7 were found by measuring the signal for five IMRT treatments and the out‐of‐field values were normalized to the last in‐field value. The uncertainties indicated on Fig. 7 represent the standard deviation of these results.

### B. development of response prediction algorithm

The signal (response), as a result of primary radiation, Rp, is linearly proportional to both the leaf pair separation (S) and the delivered MU (Figure 4, Figure 5) this can be represented in the form shown in

(1) $$R_{p}=S\times \text{MU}\times k$$

where *k* is a proportionality factor. The lateral response function allows the scatter signal at a wire A, as a result of primary radiation incident on the device above wire B, to be calculated:

(2) $$R_{sA(B)}=R_{pB}\times e^{-l}(D_{AB})$$

where $R_{sA(B)}$ is the signal at wire A as a result of the radiation incident on the PMMA above wire B; $R_{pB}$ is the response at wire B as a result of primary radiation. This is equal to the product of the MU and separation of the MLC leaf pair associated with wire B ($S_{B}$) and a proportionality factor (Eq. (1), Eq. (3)), i.e.

(3) $$R_{pB}=S_{B}\times MU\times k$$

*l* is the exponent associated with the lateral response function, and $D_{p\mathrm{AB}}$ is the distance between wire A and B.

So the total response at wire A from both primary and scatter radiation can be given by

(4) $$R_{A}=MU\times k\times \sum_{W=1}^{W=40}S_{W}\times e^{-l(D_{AW})}$$

Note that when W=A, the equation reduces to the signal from the primary radiation, so Eq. (4) includes both primary and scatter contributions to the signal. The total response matrix is then equal to the convolution of the leaf pair separation matrix multiplied by the MU and constant of proportionality:

(5) $$R=MU\times k\times (S⊗e^{-lD})$$

where *R* is the response matrix (i.e., responses for each wire for a segment), and *S* is the leaf pair separation matrix (i.e., the leaf pair separations for each MLC for a segment).

Out‐of‐field wires were given a predicted response of zero apart from the first four out‐of‐field wires, which were given predicted signals as a function of the predicted signal of the nearest wire inside the field, according to the penumbral response shown in Fig. 7.

### C. Implementation and testing

Each IMRT field, for each of the ten treatments was delivered three times. The spread in these signals, in the in‐field region, was always <1%, showing the device is stable and capable of producing consistent results.

**Figure 8:**
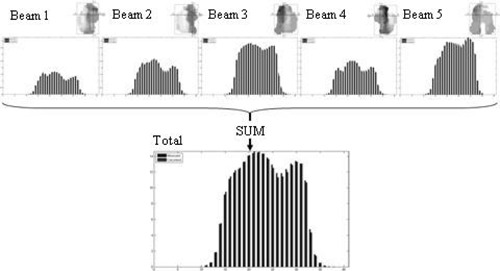
For each IMRT delivery, the individual segments were summed to give the predicted and measured signals for each wire for each beam (top). The beams were then summed to give the total signals for the treatment (bottom).

The signal for each wire and for each beam for each of the ten deliveries was predicted. These values were compared to the respective measured values, calculated from the average of three deliveries. The beam signals for each treatment were summed to give the total predicted and measured value for each treatment (Fig. 8).

**Figure 9:**
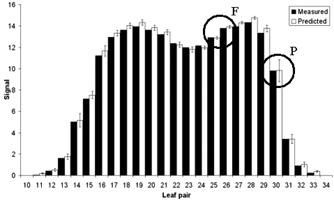
The measured and predicted DAVID signal for a full‐fraction delivery. Region F shows an area composed of mainly in‐field signals; region P is composed of more out‐of‐field signals relative to region F. The error bars are derived from the uncertainties in the model and reproducibility of the DAVID data.

**Figure 10:**
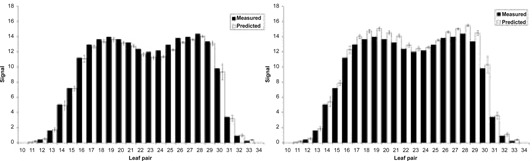
The left and the right images show the measured and predicted signals for a plan modified by −5% and +5%, respectively.

When the predicted signal for each segment was derived, the value was given an uncertainty related to its position in the field. Out‐of‐field signals were given an uncertainty, according to those derived in Fig. 7. Each in‐field signal was given a standard uncertainty of 1%. Each of these uncertainties was summed in quadrature for the beam results and these were, in turn, summed in quadrature for the full fraction results. Figure 9 shows a typical delivery; signals that were formed from more penumbral regions (P) will have a larger uncertainty than signals formed mainly from in‐field regions (F). A 5% tolerance was placed on the individual beam results; a 2.5% tolerance was placed on the full‐fraction results. To simulate a calibration error, the TPS MU was modified by ±5%; in each instance the system detected this for all ten treatments. The results for these modified deliveries are shown in Fig. 10.

## IV. DISCUSSION

The linear behavior of the response of the central wire with regard to both leaf pair separation and MU delivery (Fig. 5, Fig. 6) implies that the form of Eq. (1) is appropriate. The minimal uncertainties on the lateral response function (Fig. 4) indicate that the same lateral response function is applicable for a wide range of both separation and MU delivery.

At present the algorithm is relatively simple. This has been a deliberate choice so that, with a limited number of variables, implementation will be as simple as possible, and yet the method is sensitive enough to detect gross errors or changes in treatment delivery parameters (Fig. 10), at similar tolerance levels to diode *in vivo* dosimetry, but now checking across the whole complex delivery distribution.

The key shortcoming to the work lies in the modeling of to the out‐of‐field signals. To increase the accuracy of the model, a fundamentally different approach is being developed, based on constructing a Monte Carlo model of the DAVID. This will allow fluence maps at the level of the detection wires to be defined and an algorithm will then be designed to analytically replicate these fluence maps. The aim will be to extract the DAVID signal directly from these fluence maps.

Though the method discussed in this paper is limited to step‐and‐shoot IMRT deliveries, expansion to sliding window deliveries, using the same general approach, is possible. Plotting leaf pair separation as a function of MU delivered and integrating the result would yield a figure proportional to the primary fluence through a leaf pair; repeating this for each wire and convolving the resultant 1×40 matrix with the response function would give a predicted signal for each wire for the entire delivery.

## V. CONCLUSIONS

The DAVID device has been shown to be both stable and accurate for 50 deliveries, demonstrating that the device is a good tool for *in vivo* dosimetry and that the standard method of use,^(2)^ benchmarking to pretreatment in‐phantom verification, is both appropriate and highly accurate.

This work has developed and tested a simple predictive algorithm aimed at removing the pretreatment baseline step, and has shown this is able to predict the measured signals for the whole treatment and for individual beams to within acceptable clinical tolerances.

Use of the algorithm suggested in this paper is not as sensitive to minor leaf‐positioning errors as creating a baseline reading through actual measurement.^(2)^ Furthermore, an accuracy 5% per beam and 2.5% per treatment is not sufficient to replace the rigorous pretreatment verification that is typically done when introducing new techniques. However, once confidence has been built up in new techniques, it has been suggested that this level of rigor is no longer necessary.^(8)^ In this instance, verification of the dose with checking software coupled with signal generation using this algorithm will detect gross errors on the first treatment fraction, without the time cost of pretreatment verification on the linac (Fig. 2). Though unable to detect errors in patient setup, which in any case should be more accurately done using linked position and setup verification protocols, the suggested method (Fig. 2) would check for and detect:

- Error in treatment planning software — detected by independent checking software.
- Error in DICOM plan export — detected by independent checking software.
- Error in plan transfer, or upload to treatment delivery software — detected by the comparison of generated and measured DAVID signals. This can be done in real time, allowing the operator to stop the treatment after the first IMRT beam or, in the case of VMAT, early in the first fraction.
- Gross machine errors (incorrect calibration >5%, incorrect energy, leaf failure) —detected by the comparison of generated and measured DAVID signals.

## ACKNOWLEDGMENTS

This work was sponsored by PTW (Freiberg, Germany)

## Supporting information

Supplementary Material

Supplementary Material

Supplementary Material

Supplementary Material

Supplementary Material

## References

[1] Poppe B , Thieke C , Beyer D , et al. DAVID ‐ a translucent multi‐wire transmission ionization chamber for in vivo verification of IMRT and conformal irradiation techniques. Phys Med Biol. 2006;51(5):1237–48. 16481690 10.1088/0031-9155/51/5/013

[2] Looe HK , Harder D , Ruhmann A , Willborn KC , Poppe B . Enhanced accuracy of the permanent surveillance of IMRT deliveries by iterative deconvolution of DAVID chamber signal profiles. Phys Med Biol. 2010;55(14):3981–92. 20577038 10.1088/0031-9155/55/14/003

[3] Ash D . Lessons from Epinal. Clin Oncol. 2007;19(8):614–15. 10.1016/j.clon.2007.06.011 17656077

[4] British Institute of Radiology, Institute of Physics and Engineering in Medicine, National Patient Safety Agency , Society and College of Radiographers, The Royal College of Radiologists. Towards safer radiotherapy. London, UK: NPSA; 2008 Available from: https://www.rcr.ac.uk/docs/oncology/pdf/Towards_saferRT_final.pdf

[5] AAPM . Diode in vivo dosimetry for patients receiving external beam radiation therapy. AAPM Report No. 87. Report of Task Group 62 of the Radiation Therapy Committee. Madison, WI: AAPM; 2005.

[6] Derreumaux S , Etard C , Huet C , et al. Lessons from recent accidents in radiation therapy in France. Radiat Prot Dosimetry. 2008;131(1):130–35. 18725379 10.1093/rpd/ncn235

[7] Budgell GJ , Perrin BA , Mott JA , Fairfoul J , Mackay RI . Quantitative analysis of patient‐specific dosimetric IMRT verification. Phys Med Biol. 2005;50(1):103–19. 15715426 10.1088/0031-9155/50/1/009

[8] Nelms BE , Zhen H , Tome WA . Per‐beam, planar IMRT QA passing rates do not predict clinically relevant patient dose errors. Med Phys. 2011;38(2):1037–44. 21452741 10.1118/1.3544657 PMC3188652

