# Supplementary figures and images for: A simple model for predicting the signal for a head‐mounted transmission chamber system, allowing IMRT in‐vivo dosimetry without pretreatment linac time
Source: J Appl Clin Med Phys. 2014 Jul 8;15(4):270–9. doi: 10.1120/jacmp.v15i4.4842 (PMC5875507; doi:10.1120/jacmp.v15i4.4842)

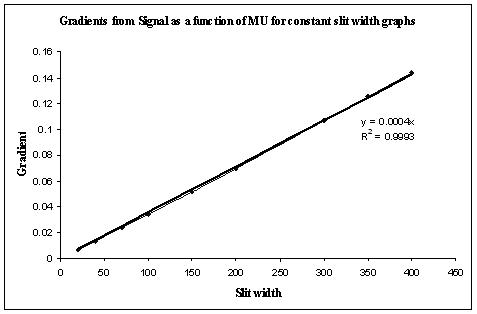

Supplement: Supplementary file 1 — Supplementary Material [file ACM2-15-270-s001.jpg]

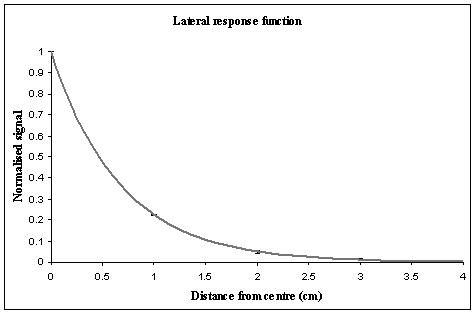

Supplement: Supplementary file 2 — Supplementary Material [file ACM2-15-270-s002.jpg]

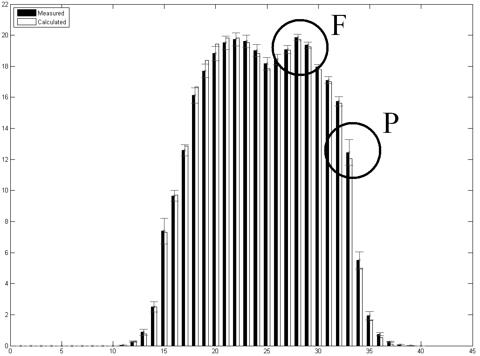

Supplement: Supplementary file 3 — Supplementary Material [file ACM2-15-270-s003.jpg]

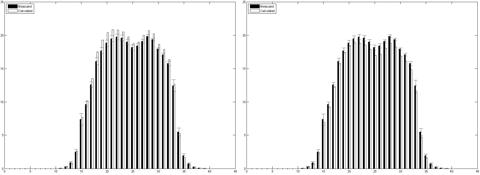

Supplement: Supplementary file 4 — Supplementary Material [file ACM2-15-270-s004.jpg]

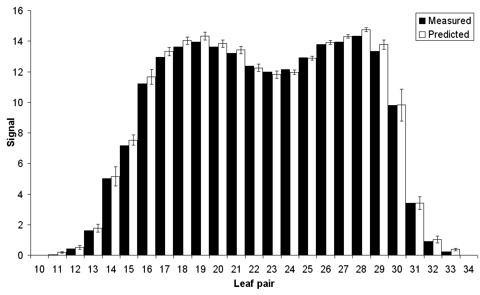

Supplement: Supplementary file 5 — Supplementary Material [file ACM2-15-270-s005.jpg]
